# Pulmonary vein flow pattern in patients with bidirectional cavopulmonary connection or Fontan circuit

**DOI:** 10.1186/1532-429X-13-S1-P202

**Published:** 2011-02-02

**Authors:** Masoud Shariat, Lars Grosse-Wortmann, Jonathan Windram, Shi-Joon yoo

**Affiliations:** 1 Hospital for Sick Children, Toronto, ON, Canada

## Background

**Figure 1:**
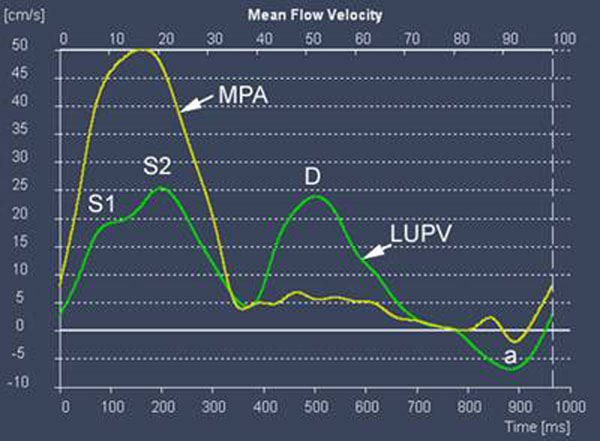


Typical flow velocities in the extraparenchymal pulmonary veins demonstrate two major antegrade flow waves: a biphasic systolic wave (S1 and S2) and a monophasic early diastolic wave (D) (Figure 1). There is often flow reversal during atrial systole (a). There is agreement that the forward diastolic pulmonary vein flow wave is caused by left ventricular relaxation and opening of the left atrioventricular valve. The origin of the pulmonary vein systolic wave, however, is not very clear, and there is considerable disagreement in the literature. S1 could be associated with a fall in pulmonary vein pressure and is compatible with a suction effect due to the combined effect of atrial relaxation and apical displacement of the atrioventricular valve plane during early systole. S2, however, could be caused by forward propagation of the right ventricular systolic pressure pulse.

## Objectives

To compare the systolic component of pulmonary vein flow in subjects with biventricular circulation and in those with functionally univentricular circulation and to determine if any of S1 or S2 are dependent on forces created by right ventricle.

## Patients and methods

We assessed pulmonary vein flow pattern in 12 cases (39 pulmonary veins) with RV-independent pulmonary circulation (Bidirectional cavopulmonary connection [BCPC] or Fontan circulation). Phase-contrast imaging of the pulmonary veins was performed on a 1.5 T MR scanner with velocity encoding set at 120 cm/s. We compared these flow patterns with the flow patterns of a control group of 10 patients (15 pulmonary veins) who had RV-dependant pulmonary circulation and underwent CMR due to other reasons (Coarctation of Aorta, Atrial Septal Defect).

## Results

**Figure 2:**
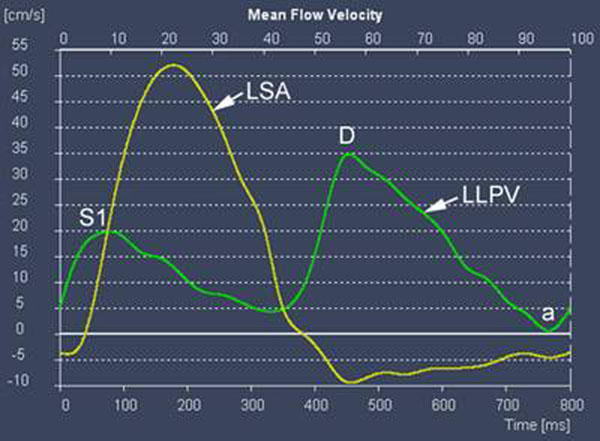


In all patients with RV-independent pulmonary circulation pulmonary vein flow curve showed a single systolic peak in early systole with the S2 peak consistently absent (Figure 2). Pulmonary vein flow pattern in the control group consistently showed early and late systolic peaks (S1 and S2) (Figure 1).

## Conclusion

This study further supports the concept that the late systolic pulse (S2) of the pulmonary vein flow curve is caused by forward propagation of the right ventricular systolic pressure pulse. It also demonstrates that the early systolic pulse (S1) is independent of the right ventricle but dependent on the left atrial and left ventricular events.

